# Incidence and dynamics of mobility device use among community-dwelling older adults in the United States

**DOI:** 10.1002/jey2.12011

**Published:** 2024-09-25

**Authors:** Xinran Liu, Sara E. Baumann, Andrea L. Rosso, Elizabeth M. Venditti, Yao Yao, Steven M. Albert

**Affiliations:** 1 Department of Behavioral and Community Health Sciences, University of Pittsburgh School of Public Health, Pittsburgh, Pennsylvania, USA; 2 Department of Epidemiology, University of Pittsburgh School of Public Health, Pittsburgh, Pennsylvania, USA; 3 Department of Psychiatry, University of Pittsburgh School of Medicine, Pittsburgh, Pennsylvania, USA; 4 China Center for Health Development Studies, Peking University, Beijing, China

**Keywords:** assistive technology, mobility device, mobility disability, user experience

## Abstract

Mobility devices are crucial in enhancing activities and participation for individuals with mobility disability, particularly among the rapidly expanding population of older adults worldwide. This paper explores patterns of mobility device use among a nationally representative cohort of community-dwelling older adults in the United States, using data from the National Health and Aging Trends Study (NHATS) waves 1–9 (2011–2019). Our descriptive analysis focuses on the characteristics of incident mobility device use, its influencing factors, and related user experiences, aligning with the NHATS late-life disability framework. Mobility devices were categorized into walking aids (WAs), wheeled and seated mobility devices (WSMDs), and mixed use of both. We identified 2,943 incidents of mobility device use among 2,591 participants, spanning 47,722 person-years in community settings, yielding an incidence rate of 61.7 per 1,000 person-years. Over half (51.3%) of mobility device use ended in 1 year, with WAs being the predominantly used (63.8%). About one-third (30.5%) of these incidents involved a change in device combinations, with a notable shift towards WSMDs and mixed use over time. We found that older adults using WSMDs or changing their device combinations were in a more vulnerable state, while those using mixed devices or changing their device combinations experienced poorer user experiences. This study advocates for the implementation of rental and recycling programs, the involvement of NGOs and professional associations, and the adoption of flexible policies responding to the dynamic patterns of mobility device use among community-dwelling older adults. It also recommends expanding services to better serve vulnerable subgroups.

## 1 | INTRODUCTION

Disability is a natural part of the human experience, impacting nearly everyone at some point in their lives.^1^ Older adults are especially vulnerable to an increased risk of disability due to an accumulation of health risks over their lifetime, including diseases, injuries, and chronic health conditions. Given the global trends of aging, it is projected that older adults will comprise a larger portion of the disability population in the upcoming decades.^1^

Over the past few decades, various definitions and conceptual models of disability have emerged, reflecting a shift from the *medical model*, which considers disability as an individual feature that requires medical treatment or intervention, to the *social model*, which perceives disability as a socially-created issue stemming from inaccessible physical environments and discriminatory attitudes that require political action.^2^ To reconcile this debate, the World Health Organization (WHO)^3^ introduced the International Classification of Functioning, Disability and Health (ICF) model. This biopsychosocial framework integrates the medical and social models, providing a more holistic understanding of disability.

In the ICF framework, disability is viewed as a dynamic and continuous state that arises from the interaction between health conditions and contextual factors, such as external environmental factors (e.g., assistive products and technology, service systems, natural and built environment, social attitudes, and political, legal and social structures) and internal personal factors (e.g., coping styles, motivation, and self-esteem).^3^

Mobility disability refers to the inability of an individual to move around their environment purposively.^4,5^ It is recognized as one of the six critical domains in the WHO Disability Assessment Schedule 2.0 (WHODAS 2.0).^5^ According to the ICF framework, mobility disability can be caused by various factors, including physical impairments, chronic health conditions, or environmental barriers, and can be either temporary or permanent.^3^

The field of assistive technology has evolved to aid individuals with disability. Assistive technology encompasses the application of organized knowledge and skills related to assistive products (e.g., devices, equipment, instruments, software), systems, and services.^6^ Specifically, mobility devices play a critical role in augmenting or replacing ambulation for individuals with limited mobility functioning.^7^ In the ICF model, these devices are considered as environmental factors and interventions designed to alleviate activity limitations and participation restrictions, thereby enhancing independence and well-being of people with mobility disability.^3^

### 1.1 | Prevalence of mobility disability among U.S. older adults

Mobility requires complex body movements that depend on the collaboration of multiple body systems: the skeletal and muscular systems provide the locomotive structure; the nervous system is responsible for information gathering, movement planning, and coordination of structural components; and the cardiovascular and respiratory systems transport energy, supporting force generation and information processing.^8,9^ Health conditions impacting any of these systems can lead to a decline in mobility functioning and result in mobility disability. The 2011 National Health and Aging Trends Study (NHATS)^10^ highlights several leading health conditions that contribute to mobility disability among U.S. older adults, including hip fracture, dementia, vision impairment, stroke, heart disease, osteoporosis, diabetes, pain, and arthritis.^11^

In the United States, mobility disability is the most frequently reported type of disability among older adults. A study analyzing the 2016 Behavioral Risk Factor Surveillance System data revealed that 41.7% of U.S. adults aged 65 and over reported having some form of disability, with 64.5% of these individuals specifically experiencing mobility disability.^12^ This suggests that roughly one in every four older adults in the United States faces challenges with mobility. For a more stringent measure of mobility impairments using the Nagi’s physical disability scale^13^ in the NHATS^10^ data, the prevalence of mobility disability among U.S. adults aged 65 and over was 83.1% in 2011% and 84.7% in 2015. The prevalence of mobility disability among older people in the U.S. is higher in certain demographic groups: women, racial minorities, individuals with low economic status, and individuals residing in the Southern region.^12^

### 1.2 | History and types of mobility devices

The history of mobility devices stretches back thousands of years, as evidenced by artifacts such as a crutch-like stick buried with an Egyptian mummy from the 25th Dynasty (744–656 BC),^14^ a cane held by an Italian Franciscan friar in a 16th century wooden painting,^15^ and the first wheelchair appeared in an ancient Chinese stone engraving dated AD 525.^16^ The first self-propelled wheelchair was developed in Italy in the mid-15th century, but it was not until 1814 that John Joseph Merlin (1735–1803) designed the prototype of the modern wheelchair used today.^16^ In the mid-20th century, driven by ongoing wars, polio epidemics, and the aftermath of the Thalidomide tragedy, engineering research and development of mobility devices flourished, with increased attention on power wheelchairs.^17^ Today, research on mobility devices has broken new ground, with new designs of exoskeletons^18^ and new technologies for wheelchair control and monitoring, such as brain-computer interfaces^19^ and route navigation.^20^

Despite these technical advancements and the increasing variety of mobility devices, global access to these devices remains limited. To address this issue, the WHO introduced the Priority Assistive Products List (APL) in 2016, aiming to enhance access to affordable and high-quality assistive products, including mobility devices, worldwide.^6^ The mobility devices listed in the APL can be categorized into walking aids (WAs) and wheeled and seated mobility devices (WSMDs). Walking aids, or ambulation aids, are designed to support balance and/or weight-bearing through the legs, facilitating walking for individuals with impaired mobility. This category includes canes, crutches, walking frames, and rollators, which differ in their general features, such as the requirement of using one or both arms for support, and their assistive potential.^6^ Wheeled and seated mobility devices, including manual wheelchairs, power wheelchairs, and scooters, share the common feature of enabling wheeled mobility while remaining seated, offering users greater ease and independence to move around.^21^

### 1.3 | Use of mobility devices among U.S. older adults

In the 2011 NHATS study, 8.5 million (24.1%) of U.S. older adults reported using mobility devices in the last month, with 3.2 million (9.3%) using more than one device.^11^ Specifically, 16.4% used canes, 11.6% used walkers (including crutches, walking frames, and rollators), 6.1% used manual or power wheelchairs, and 2.3% used scooters.^11^

There was a fourfold increase in the prevalence of mobility device use from 6.2% at the beginning of the millennium to 24.1% by 2011.^22^ However, this prevalence stabilized in this population between the 2011 and 2015, remaining at 25.0%, which represents approximately 15.7 million users in 2015 (NHATS). The use of mobility devices (of any type) was notably more common among older adults who were of advanced age, female, racial minorities, had lower education levels, and lower household incomes.^11,23,24^ Additionally, mobility device use was more prevalent among older adults who were widowed and living alone.^23^ Regarding health characteristics, the use of mobility devices was associated not only with specific diseases and injuries that lead to mobility disability, but also with impaired balance and coordination, perceived poor health status, obesity, and a higher number of chronic conditions.^11,23^

### 1.4 | Dynamic patterns of using mobility devices

The use of mobility devices among older adults is a dynamic process. In the 2011 NHATS study, 7.9% of U.S. older adults who were not using any mobility devices at baseline began to use them 1 year later, while 15.7% of those who were using mobility devices discontinued their use after 1 year.^11^ Notably, these patterns shift significantly with age. Older age groups are more likely to start using mobility devices and less likely to discontinue their use. For instance, an observational study across five European countries revealed that the 1-year cumulative incidence rate of starting to use mobility devices was 11.7% for people aged 75–84% and 18.8% for those aged 80–89, while the rates of stopping their use were 18.1% and 7.8%, respectively.^25^ Further, in a 5-year follow-up study of the oldest-old in Sweden, 74% of older adults who were not using mobility devices at age 85 started using one by age 90, while only 1.4% of those who were using mobility devices at age 85 had stopped using them by age 90.^26^

The dynamic pattern of mobility device use involves not only the initiation and the cessation of use but also changes in the devices used over time. The trajectories of incident mobility device use can be categorized into as many as seven types, as observed by Demers et al.^27^ in a 6-month follow-up of 139 patients (age 64.2 ± 16.2 years) who procured a mobility device upon discharge from the hospital. These categories were based on whether the mobility devices procured upon discharge were continuously used and if a second device, either of the same or a different type, was introduced. In another cohort study of 101 older patients (age 70+ years) who were discharged with a walking aid after a hip fracture, Thomas et al.^28^ reported that 82% of these patients made their own decisions to change their walking aids, typically within an average of 2 months post-discharge. However, a notable concern is that 32% of these changes were deemed inappropriate by the research physiotherapist, potentially posing safety risks.^28^

### 1.5 | Factors influencing the use of mobility devices among older adults

Utilizing the social-ecological model,^29^ we summaized various factors influencing the use of mobility devices among community-dwelling older adults. These factors are organized according to the following levels: individual, interpersonal, organizational, community, and societal.

#### 1.5.1 | Individual factors

In addition to health conditions leading to mobility disability and demographic and socioeconomic characteristics influencing mobility device use, several other individual factors significantly impact older adults’ preferences for mobility devices. These include older adults’ *attitudes towards using mobility devices*, the *functional capacities and skills* necessary for operation, and their *user experience of mobility devices*.

Both positive and negative *attitudes towards using mobility devices* exist among older users,^30^ with those experiencing higher mobility disability showing more positive attitudes.^31^ Positive attitudes such as self-efficacy, or the belief in one’s abilities to use mobility devices effectively, which can lead to better device skills and performance^32,33^; and autonomy, or the perceived ability to move freely and independently, is crucial for older adults, influencing their likelihood of using mobility devices when facing functional decline.^34,35^ Resistance to using mobility devices often stems from their associations with aging and disability stereotypes, leading to embarrassment and stigma when using the devices.^35,36^ Attitudes towards mobility devices generally shift from negative to positive over time, through stages from “seeing the device as a sign of decline” to “accepting it as part of the body”.^37(p537)^ Embracing a new self-image with a mobility device is beneficial, as it can enhance device users’ independence and facilitate activity and participation.^38–40^

Specific *functional capacities* are necessary for the independent use of mobility devices. Cognitive functions are essential for the safe use of walking aids,^41–44^ while more advanced cognitive functions are required for wheelchairs and scooters.^45,46^ Physical functions, such as knee extension strength, are crucial for walking aid users,^47^ while manual wheelchair users need sufficient trunk and upper-extremity muscle strength.^48,49^ Older manual wheelchair users often switch to motorized wheelchairs or scooters due to propelling difficulties and declining aerobic capacity,^45,50^ which require fine motor abilities^51^ and grip strength for control.^52^ Visual functions, including visual acuity and visual field, significantly affect navigation and driving performance of mobility device users.^53–56^ Mastery of necessary skills is key to enhancing independent use and safety with mobility devices. While walking aids are relatively easier to learn,^57^ wheeled and seated mobility devices present a greater challenge due to unfamiliar hand-propelling/controlling gestures and advanced skills.^33,58^ These requirements pose challenges for older mobility device users, as their functional capacities decline alongside the loss of mobility and increased reliance on mobility devices.

The *user experience of mobility devices* varies depending on their design and intended use. A cross-sectional study by Hoenig et al.^59^ compared the user experience of rollators, manual wheelchairs, and power wheelchairs among community-dwelling older adults. The study found that rollators offered the fastest speed and fewest collisions in completing both indoor and outdoor tasks. In contrast, manual wheelchairs were associated with a significant increase in fatigue and pain, while power wheelchairs, although having the most outdoor collisions and being the slowest indoors, showed the least increase in fatigue among users. Such variations in user experience are crucial, as negative experiences can lead to abandonment of mobility devices by older adults who need them.^60^

#### 1.5.2 | Interpersonal factors

While mobility devices reduce dependence on human assistance,^61^ many older users still need help with mobility, especially those using wheelchairs. According to the 2011 NHATS, 45.2% of U.S. older mobility device users received personal assistance for mobility, compared to 24.8% among non-users.^62^ Caregivers, often spouses, need to develop specific skills for maneuvering these devices and performing transfers. These caregivers, who are also aging themselves, face concerns about their diminishing capacities and the increased hazards of performing these tasks.^38,63^ However, the use of motorized mobility devices can alleviate their burden of pushing and transferring,^64^ and specialized trainings for caregivers can enhance their wheelchair skills, confidence, and the comfort and safety of the older care recipients.^65,66^

Not only caregivers, but also other individuals within an older mobility device user’s social network, can significantly impact their experience with their mobility devices. Opinions from close connections, including friends, family, and healthcare providers, influence older adults’ decisions to use mobility devices.^30,35^ Support from these groups, particularly from peer role models who are experienced mobility device users, can help older individuals regain autonomy and foster greater acceptance.^67^ Additionally, assistant animals, such as mobility assistant dogs, provide not only physical benefits, like performing various tasks and assisting in propulsion to reduce fatigue and pain in wheelchair users,^68,69^ but also positive psychological impacts, enhancing users’ acceptance of their new self-images with mobility devices.^70,71^

#### 1.5.3 | Organizational factors

The provision of mobility devices and related services is essential for addressing the growing demands of older adults, as their unmet needs represent the fastest-growing segment globally.^72^ In response, the WHO^73^ established an eight-step service delivery process, widely adopted worldwide,^74–76^ comprising referral and appointment, assessment, prescription, funding and ordering, product preparation, fitting, user training, and follow-up, maintenance and repairs. This system hinges on the collaboration of key stakeholders: clients who are in need of mobility devices, providers including health and rehabilitation workers and volunteers, suppliers such as manufacturers and vendors, and payers like health insurance and public support systems.^77^ Their collective efforts determine the service delivery’s appropriateness, timeliness, and client satisfaction, to further facilitate clients’ activities and participation.

#### 1.5.4 | Community factors

The community built and natural environments play pivotal roles in the ICF model, due to the reciprocal capability-demand relationship between humans and the physical environment.^78^ Older adults with lower functional capacity are more affected by environmental demands, leading to poorer task performance, reduced activities, and limited participation.^79^ The Facilitators and Barriers Survey for People with Mobility Limitations Version 2^©^ (FABS-Mv2) evaluates the accessibility and usability of community environments for mobility device users,^80^ categorizing influential factors into domains like home environment built features, community environment built and natural features, usability of community building sites and public restrooms, and usability of personal and community transportation. Although community accessibility compliance is improving, especially in recently constructed buildings and outdoor facilities,^81^ higher-demand environments like transport facilities, building entrances, restrooms, stairs and escalators continue to present challenges.^82^ These challenges often increase the dependence of older mobility device users on *personal assistance*.^38^

To counter environmental barriers and enhance accessibility and usability, implementing barrier-free designs is essential, particularly in areas where older mobility device users spend most of their time. *Home modifications* can lower environmental demands, reduce the need for personal assistance, and encourage independent living.^83^ The use of mobility devices and home modifications involve a mutual adjustment process,^84^ where device users learn to use different devices for varying environmental demands, such as using a wheelchair at home and supplementing it with a walker in more confined spaces like the bathroom.^85^

#### 1.5.5 | Societal factors

Evolutionary theories of disease avoidance suggest that humans have evolved a set of universal psychological processes to detect environmental cues of pathogens or infectious diseases, which initiate cognitive and emotional responses such as disgust and avoidance behaviors.^86^ This mechanism also extends to reactions towards individuals with noncontagious physical impairments, potentially resulting in implicit and unconscious biases against people with disabilities.^87^ Research by Rohmer and Louvet^88^ indicates that disability is often perceived as a more salient social category than gender or race/ethnicity. This results in a structural asymmetry where disability frequently becomes a prominent descriptor, in contrast to able-bodiedness, which is seldom referenced. This imbalance in social categorization perpetuates disability stereotypes, such as the schematic of a wheelchair user becoming an international symbol of disability.^89^ Such societal attitudes and stereotypes can make mobility device users feel marginalized and unwelcome in public spaces,^30,90,91^ potentially discouraging their use of mobility devices to go out.^92^

However, the plasticity of social attitudes offers an opportunity for improving disability awareness.^93^ Promoting disability visibility in popular culture, such as through comics, can positively shift public perceptions.^94^ Immersive interventions and experiential learning involving the use of mobility devices have proven effective in fostering understanding among healthcare students, professionals, and other stakeholders involved in mobility device service delivery.^95–98^ Positive social attitudes, exemplified by efforts to replace the access icon of “cyborg of wheelchair and person” with an active wheelchair rider,^99(p3)^ significantly influence both the experiences of mobility device users and the development and implementation of relevant policies and guidelines.^100,101^ A significant milestone is the Convention on the Rights of Persons with Disabilities (CRPD) established by the United Nations (UN) in 2006, the first human rights treaty focused on the rights of individuals with disabilities, aiming to ensure their full participation in society and equal access to all areas of life.^102,103^ As of 2023, 184 UN member states (94.8%) have ratified the CRPD, though only 60% of member states have enacted corresponding national disability laws, such as the Americans with Disabilities Act^104^ in the United States. The CRPD, along with national laws, lays the groundwork for political commitment and legislation framework, further facilitates the allocation of funding and the development of implementation systems.^72^ This comprehensive approach aims to enhance access to mobility devices and improve environmental adaptations at both organizational and community levels, ultimately promoting the engagement and inclusion for individuals with mobility disability.

### 1.6 | National health and aging trends study and research on mobility device use

The National Health and Aging Trends Study (NHATS) is a longitudinal cohort study initiated in 2011 to evaluate health, functionality for independent living, and quality of life of Medicare beneficiaries aged 65 and older in the United States.^10^ This cohort extensively collects data on assistive technology, including mobility devices. However, there is a notable gap in research on mobility devices using NHATS data. Most past research has applied a cross-sectional design,^24,62,105–111^ which may neglect the dynamic changing patterns of mobility device use over time. Other longitudinal studies on mobility devices using NHATS data either had short follow-up durations,^11,112^ focus exclusively on one specific type of mobility device,^113^ or involved mobility devices as just one element of a broader category of assistive technologies.^114,115^

## 2 | OBJECTIVES OF THE PAPER

Mobility device use is prevalent among U.S. older adults, and plays a crucial role in maintaining their activity, participation, and independent living in communities. However, the patterns of mobility device use among this demographic group have not been comprehensively explored in a nationally representative sample, particularly over an extended longitudinal period and across all types of mobility devices. This paper aims to detail the trajectories of mobility device use among community-dwelling older adults in the United States. We will identify the distinctive features of mobility device use within this age group. Additionally, we will describe the factors that influence mobility device use and the user experiences related to these devices in community settings. This exploratory descriptive study aims to provide deeper insight into the patterns of mobility device use among community-dwelling older adults, how the mobility device use is influenced by the characteristics of the users, and how these patterns affect the daily lives of these individuals.

## 3 | METHODS

### 3.1 | Data source

This study analyzed data from the National Health and Aging Trends Study (NHATS).^10^ Utilizing a stratified, multistage sampling method, the NHATS achieves national representativeness by encompassing 96% of this demographic. The initial cohort, established in 2011, included 8,245 older adults, with intentional oversampling of individuals aged 85 and older and Black non-Hispanic adults to ensure adequate representation of these subgroups.^116^ To sustain the cohort’s representativeness, an additional sample of 4,182 participants was incorporated into the study in 2015.^10^

The NHATS cohort collects comprehensive data annually through in-home interviews, which consist of self-reported questionnaires completed by the sample person or their proxy, alongside objective physical performance assessments. The scope of this study includes data from waves 1 to 9, covering the years 2011–2019, a period deliberately chosen to preclude the potential impacts of the COVID-19 pandemic. The inclusion criteria for our analysis are participants who reported new (incident) mobility device use in community settings—specifically, those who did not report using any mobility devices in one wave, but did report use in a subsequent wave. Continuous use of any mobility device is tracked from the years 2012 through 2019.

### 3.2 | Conceptual framework

**FIGURE 1:**
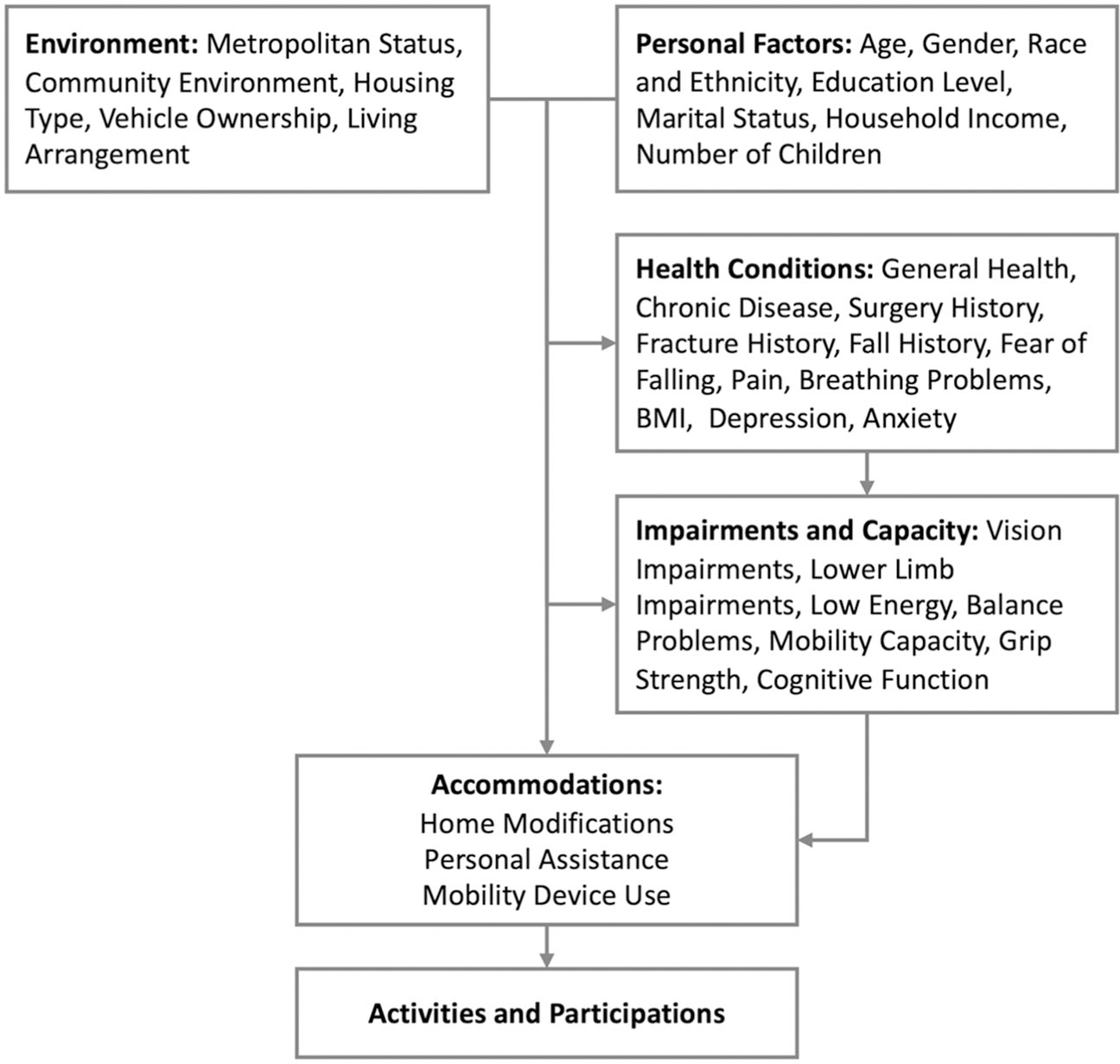
Conceptual framework of using mobility devices in community settings. Adapted from “Adopting the ICF language for studying late-life disability: a field of dreams?”^117^ Copyright 2009 by The Gerontological Society of America.

Figure 1 illustrates the conceptual framework that guides data collection and analysis in this study. It is adapted from Freedman’s NHATS late-life disability framework,^117^ which itself is informed by the WHO’s ICF model.^3^ The NHATS framework maintains the comprehensive scope of the ICF model, while highlighting the significance of *accommodations*—defined as behavioral responses to changes in functional capacity.^117^

In the context of community-dwelling older adults, declines in mobility capacity are addressed through *accommodations* such as using mobility devices, obtaining personal assistance, and making home modifications (Figure 1). These *accommodations* are influenced by a spectrum of upstream factors, as outlined in the framework, including personal factors, environment, health conditions, body impairments, and functional capacity (Figure 1). The integration of these *accommodations* plays a critical role in shaping the daily activities and social participation of older adults with mobility disability, ultimately impacting their health-related quality of life and overall well-being.

### 3.3 | Key variables

The variables of interest for this study, as depicted in Figure 1, span the upstream domains that influence the use of mobility devices among community-dwelling older adults. These upstream variables were collected at each incident of mobility device use, specifically one wave before the wave where participants began reporting the use of any mobility devices in community settings. The primary focus of this study is on the *incident mobility device use*, with a particular emphasis on the user experience. These mobility device use-related variables were followed up annually throughout the duration of each incident. The domains and key variables collected in the NHATS cohort are outlined below.^118^

#### 3.3.1 | Mobility device use

The utilization of mobility devices was based on participants’ responses to the question, “In the last month, have you used a cane, walker, wheelchair, or scooter, yes or no?”. Individuals reporting the use of any one of these devices—cane, walker (including walking frames with or without wheels and rollators), wheelchair (both manual and power), or scooter—were classified as mobility device users. The *types of mobility devices* were categorized based on the combination used by participants: *walking aids* (WAs), encompassing canes and walkers; *wheeled and seated mobility devices* (WSMDs), including wheelchairs and scooters; and a *mixed* category, comprising both WAs and WSMDs. *Incident mobility device use* was identified when participants, initially not using any devices, reported their use in subsequent visits. The *duration of incident mobility device use* was recorded as a continuous variable ranging from 1 to 8 years. Furthermore, any changes in mobility device combinations during the incident were captured and summed up as the *number of mobility device combination changes*, with possible values ranging from 0 to 7. The study further examined the user experience for each incident of mobility device use through various measures: the *frequency of using mobility device indoors/outdoors* and the *frequency of holding onto walls/furniture when moving around at home* (both ordinal variables with a range from 1 = never to 5 = every time), the *difficulty of using mobility devices indoors/outdoors* (ordinal, ranging from 1 = none to 4 = a lot), and *costs on assistive products* (ordinal, ranging from 1 = less than $100 to 4 = more than $1,000), encompassing participants’ annual total expenditure on assistive products aiding with vision, hearing, mobility, and other activities of daily living.

#### 3.3.2 | Personal factors

*Age* was recorded as a continuous variable for participants before the onset of *incident mobility device use*, starting at 65 years. *Gender* was recorded as a binary variable, with 1 representing female. *Race and ethnicity* were categorized as 1 = White, non-Hispanic; 2 = Black, non-Hispanic; 3 = other, non-Hispanic; 4 = Hispanic; and 5 = multiracial. *Education level* was classified into 1 = less than high school, 2 = high school diploma, 3 = some college and Bachelor’s, and 4 = graduate education. These variables were established at the NHATS cohort baseline, collected either in 2011 for the original sample or in 2015 for the additional sample.

*Marital status* was noted as 1 = married or with a partner, 2 = single, and 3 = widowed. *Household income* was a categorical variable grouped by quartiles. The *number of children* was an ordinal variable, coded as 1 = no children, 2 = single child, and 3 = two or more children. These variables were gathered before the participants began using mobility devices.

#### 3.3.3 | Environment

The living environment of participants, before the onset of *incident mobility device use*, was characterized by several variables. *Metropolitan status* was noted as a binary variable, with 1 indicating a residence in metropolitan area. *Community environment* was a synthesized continuous variable measuring participants’ perception of their community (neighborliness, helpfulness, and trustworthiness) on a scale from 1 to 9, where higher scores suggest a more amicable community environment. *Housing type* was categorized by trained staff during in-home interviews and included free-standing single house, house attached to others (such as row house, townhouse, duplex, triplex, or triple decker), mobile home or trailer, multi-unit building, and other. *Vehicle ownership* was recorded as a binary variable, denoting the presence of any vehicle in the household. *Living arrangement* was classified into four categories: living alone, living with a spouse/partner, living with a spouse/partner and others, and living with others.

#### 3.3.4 | Health conditions

The study collected data on participants’ mobility-related health conditions reported before their use of mobility devices. Participants’ *general health* was assessed using a question from the Centers for Disease Control and Prevention’s health-related quality of life (CDC HRQOL-4),^119^ which is an ordinal variable ranging from 1 (excellent) to 5 (poor). *Chronic disease* captured the count of mobility-related health conditions reported by participants, including heart diseases, arthritis, osteoporosis, lung diseases, stroke, dementia or Alzheimer’s, and cancer, on a scale from 0 to 7. *Surgery history* was quantified by the number of surgeries that could affect participants’ mobility (knee, hip, spine, and heart), with values ranging from 0 to 4. *Fracture history* at the hip or other sites after the age of 50 was recorded using binary variables. *Fall history* recorded the number of falls experienced in the past 12 months as an ordinary variable (0 = no fall, 1 = one fall, 2 = multiple falls). Symptoms such as *fear of falling, pain,* and *breathing problems* were coded as ordinal variables (0 = no symptoms, 1 = symptoms present, 2 = symptoms limit activities). *Body Mass Index* (BMI) was derived from participant’s self-reported height and weight and categorized into underweight (<18.5 kg/m^2^), normal (18.5–24.9 kg/m^2^), overweight (25.0–29.9 kg/m^2^), and obesity (≥30 kg/m^2^). *Depression* was assessed with the Patient Health Questionnaire-2 items (PHQ-2),^120^ and *anxiety* was measured using the Generalized Anxiety Disorder-2 items (GAD-2),^121^ both ranging from 0 to 6, where higher scores indicate a greater risk.

#### 3.3.5 | Impairments and capacity

Participant’s mobility-related body impairments and functional capacity were evaluated before the use of mobility device. *Vision impairments* was self-reported by participants and coded as an ordinal variable (0 = no eyesight impairments, 1 = either near-sight or far-sight impairments, 2 = both near-sight or far-sight impairments). *Lower limb impairments*, *low energy*, and *balance problems* were similarly coded as ordinal variables (0 = no impairments, 1 = impairments present, 2 = impairments limit activities). The assessment of participants’ *mobility capacity* utilized both subjective and objective measures. The subjective component was derived from Nagi’s physical disability scale,^13^ containing a series of four binary questions that asked if, in the last month, participants could independently: (1) walk six blocks, or about half a mile; (2) walk three blocks; (3) walk up 20 stairs, approximately two flights; and (4) walk up 10 stairs. Responses were summed to create a score ranging from 0 to 4, with higher scores indicating more severe mobility impairments. The Short Physical Performance Battery (SPPB)^122^ provided an objective measure of *mobility capacity*, evaluating balance, gait, and lower limb strength, with scores from 0 to 12 points—higher scores reflected better performance. *Grip strength* was quantified using a dynamometer, where participants were instructed to squeeze as hard as they can. The average and maximum grip strength of two attempts was calculated. *Cognitive function* was assessed with the clock drawing test,^123^ scoring from 0 to 5, with higher scores reflecting better cognitive function.

#### 3.3.6 | Other accommodations

*Home modifications* refers to environmental changes made to facilitate the use of mobility devices within the home and community. This includes features such as stairs at the entrance and stairs in the home, both recorded as binary variables where 1 signifies their presence and 0 indicates absence. Bathroom modifications were quantified as a continuous variable, with a range from 0 to 4, reflecting the number of modifications made including bath grab bars, shower seats, raised toilets, and toilet grab bars. *Personal assistance* was evaluated based on the level of support provided to participants for indoor and outdoor mobility. This was coded as an ordinal variable: 0 indicates no mobility assistance, 1 for assistance in either an indoor or outdoor setting, and 2 for assistance in both settings.

### 3.4 | Analysis strategy

To delineate the characteristics of *incident mobility device use*, we restructured the data to accommodate the temporal dynamics of use. For each incident, ordinal variables were averaged across both the four categories of devices and the duration of use. These variables include the *frequency of using mobility devices indoors/outdoors, the difficulty of using mobility devices indoors/outdoors, the frequency of holding onto walls/furniture when moving around at home,* and *costs on assistive products.*

For stratified analysis, we aggregated data on the *types of mobility devices* for each incident period, summarizing the various categories of mobility devices used in each incident. We also summed up the *number of mobility device combination changes* per incident, subsequently categorizing them into three strata for analysis: no change, one change, and two or more changes. Comparative analyses of variables from upstream domains and those related to the user experience of mobility device were conducted based on these two stratification indicators, by *types of mobility devices* and the *number of mobility device combination changes*, respectively.

Descriptive statistics of key variables were reported as means with standard deviations (SDs) for continuous and select ordinal variables, and as counts with percentages (%) for categorical variables. In the stratified analyses, we employed ANOVA^124^ for continuous and ordinal variables, and Pearson’s Chi-squared test^125^ for categorical variables. Missing data were omitted from the statistical analysis due to their minimal percentages.

Although the NHATS cohort was originally designed to use survey weights for representing national statistics,^126^ these weights were not applied in this analysis. This decision was based on the time origin being set at the beginning of incident mobility device use in this study, whereas the participants’ survey weights were established according to calendar time in the NHATS cohort. Given the time-sensitive nature of survey weights and their value assignment across different calendar times, they could not be uniformly applied in this reorganized cohort.^127^

All statistical analyses were performed using STATA 18.0.^128^ For data visualization, the patterns of *incident mobility device use* were illustrated using horizontal line plot,^129^ while transitions in mobility device combinations were depicted with Sankey diagram.^130^

## 4 | RESULTS

### 4.1 | Characteristics of incident mobility device use among older adults in community settings

Throughout the follow-up of 12,427 NHATS participants from 2011 to 2019, we accumulated an observation of 47,722 person-years within community settings, with an average follow-up duration of 3.8 ± 3.0 years per participant. Among this cohort, 2,591 participants reported 2,943 *incidents of mobility device use*, yielding an incidence rate of 61.7 per 1,000 person-years. The average age of these device users was 80.3 ± 7.2 years, with 57.7% being female, 70.2% White, 20.0% Black, and 5.7% Hispanic. A notable proportion of these individuals was either single (15.5%) or widowed (38.4%), had completed high school or less (52.4%), and had two or more children (80.1%). The majority resided in metropolitan areas (80.0%), in a free-standing single house (74.4%), and owned a vehicle (80.6%), with 34.6% living alone.

**FIGURE 2:**
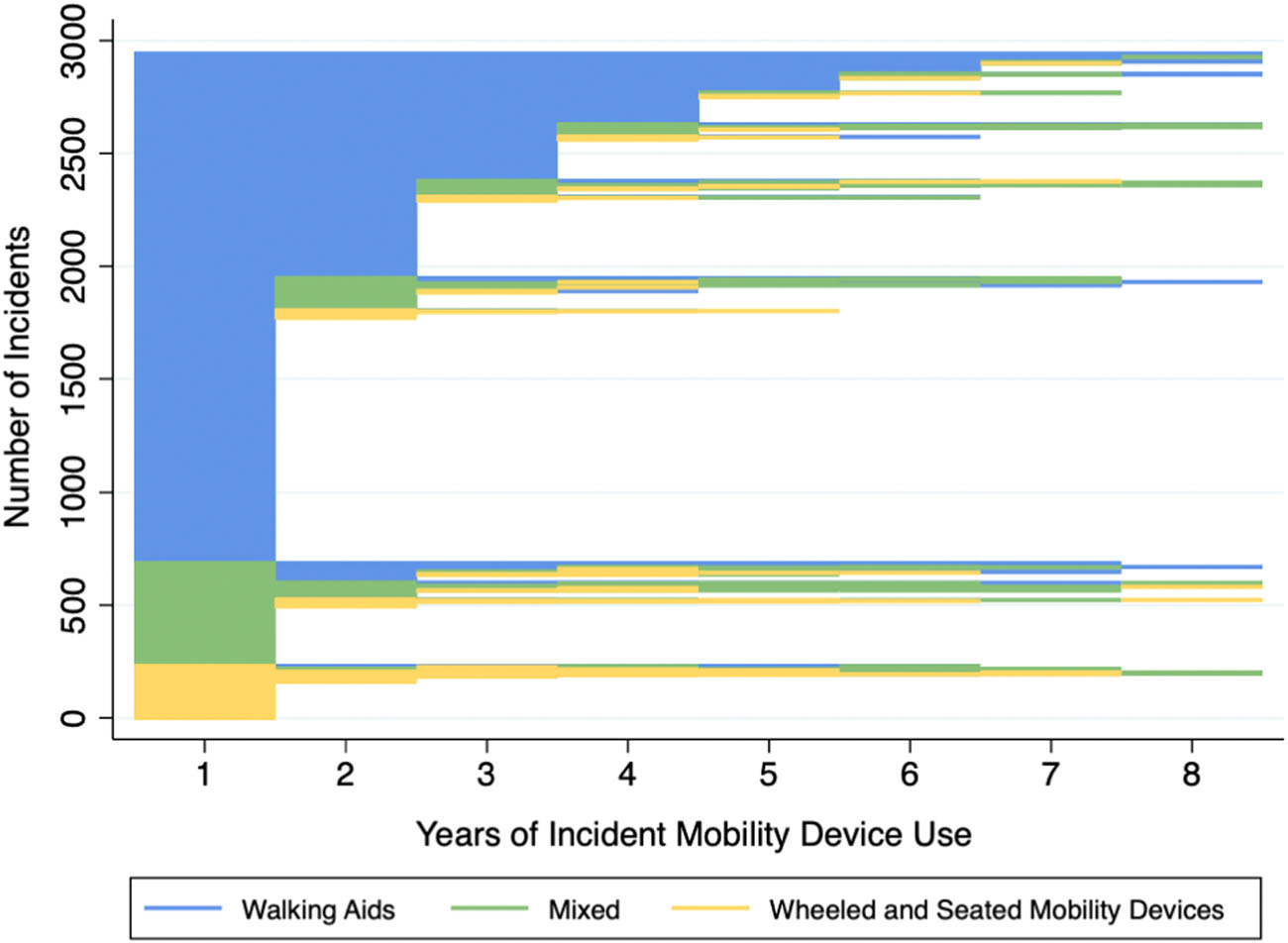
Incident mobility device use in community setting, by mobility device types.

Figure 2 visualizes all 2,943 *incidents of mobility device use*, with each horizontal line representing an individual incident. The colors help distinguish the *types of mobility devices* being used at certain periods: blue for walking aids (WAs), green for mixed device use, and yellow for wheeled and seated mobility devices (WSMDs). The length of each horizontal line indicates the *duration of incident mobility device use*. The plot is sorted by the *types of mobility devices* for enhanced readability. The WAs were predominant (63.8%), followed by mixed device use (29.7%) and WSMDs (6.6%). The most prevalent *mobility device combinations* were cane only (44.3%), cane and walker (15.7%), walker only (15.1%), and walker with wheelchair (8.3%). The *duration of incident mobility device use* showed a highly right-skewed distribution, with over a half (51.3%, or 1,510 incidents) ending within 1 year, and less than a fifth (18.6%, or 547 incidents) persisting beyond 3 years. The mean duration was 2.2 ± 1.6 years, with a median of 1 year and an interquartile range (IQR) of 1–3 years.

**FIGURE 3:**
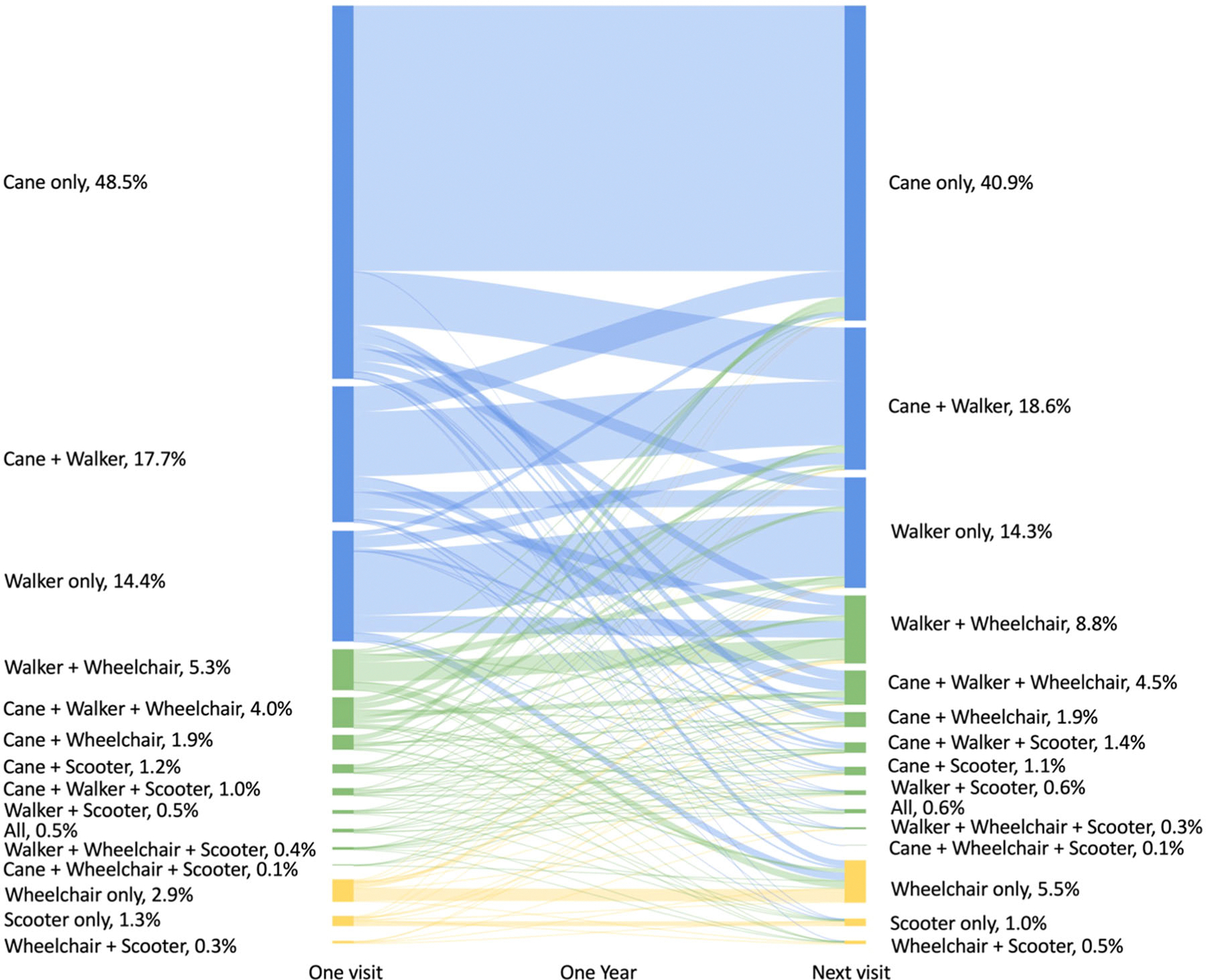
Changes in mobility device combinations in one year.

We also observed frequent *changes in mobility device combinations* over time in Figure 2. Among 898 incidents, there were 1,456 changes, averaging 1.6 ± 1.0 changes per incident. This accounts for about one-third (30.5%) of all recorded incidents. Figure 3 summarizes all transitions in mobility device combinations between two consecutive visits 1 year apart, excluding transitions during the initial and final visits of each incident. Out of a total of 3,442 transitions, we observed a notable shift in device combinations over 1 year. There was a decrease in the use of WAs from 80.5% to 73.8%, while the proportions of mixed device and WSMDs use increased from 14.8% to 19.2% and from 4.6% to 7.0%, respectively. Interestingly, the most prevalent *changes in mobility device combinations* indicated both an increased dependence on these devices (e.g., from cane only to cane and walker, 6.9%; from walker only to walker and wheelchair, 2.2%) as well as decreased dependence (e.g., from cane and walker to cane only, 3.4%; from cane and walker to walker only, 2.1%).

### 4.2 | Baseline characteristics of older mobility device users in community settings

To gain deeper insights into the initial status of community-dwelling older adults before they began using mobility devices, we analyzed their baseline characteristics. This analysis was stratified according to two primary aspects of their device use: the *types of mobility devices* and the *changes in mobility device combinations*.

Table 1 presents the baseline characteristics of these older individuals, stratified by the *types of mobility devices* they would later use. Notable differences emerged among users of WAs, mixed device users, and those using WSMDs. In terms of *personal factors* and *environment*, WSMDs users were generally older (81.3 ± 8.0 years, compared to WAs at 79.8 ± 7.0 and Mixed at 81.1 ± 7.4, *p* < .001), more likely to live in a free-standing single house (77.7%, compared to WAs at 73.5% and Mixed at 75.7%, *p* < .001) and more often living with others (29.5%, compared to WAs at 18.2% and Mixed at 22.1%, *p* < .001). Moreover, mixed device users showed a higher tendency to be widowed (41.6%, compared to WAs at 37.0% and WSMDs at 37.8%, *p* < .05).

The *health conditions* of WSMDs users were notably poorer, with worse general health (3.2 ± 1.1, compared to WAs at 2.9 ± 1.0 and Mixed at 3.1 ± 1.0, *p* < .001) and a higher burden of chronic diseases (2.2 ± 1.2, compared to WAs at 1.9 ± 1.2 and Mixed at 2.0 ± 1.3, *p* < .01). WSMDs users also reported more prevalent fear of falling (17.8%, compared to WAs at 10.4% and Mixed at 14.9%, *p* < .001) and breathing problems (18.7%, compared to WAs at 11.5% and Mixed at 16.5%, *p* < .001) that limited their activities. Furthermore, there were more mixed device users reporting multiple falls before device use (15.5%, compared to WAs at 10.7% and WSMDs at 10.9%, *p* < .001). Overall, both WSMDs and mixed device users had higher levels of depression (PHQ-2 scores: WSMDs 1.2 ± 1.5 and Mixed 1.2 ± 1.5, compared to WAs at 1.0 ± 1.3, *p* < .05) and anxiety (GAD-2 scores: WSMDs 1.1 ± 1.6 and Mixed 1.1 ± 1.5, compared to WAs at 0.9 ± 1.3, *p* < .001) than WAs users. However, WAs users reported a higher prevalence of pain that limited their activities (30.3%, compared to Mixed at 29.7% and WSMDs at 27.5%, *p* < .05) and a higher body weight (BMI Overweight + Obesity: WAs 68.2%, compared to Mixed at 62.5% and WSMDs at 52.3%, *p* < .001).

Regarding body *impairments* and functional *capacity*, WSMDs users were in a poorer condition. They reported higher levels of vision impairments (0.4 ± 0.6, compared to WAs at 0.2 ± 0.4 and Mixed at 0.3 ± 0.5, *p* < .001), poorer balance function limiting their activities (25.4%, compared to WAs at 17.5% and Mixed at 22.7%, *p* < .001), worse mobility capacity in both subjective (Self-reported mobility impairments: WSMDs 1.7 ± 1.7, compared to WAs at 1.1 ± 1.4 and Mixed at 1.5 ± 1.5, *p* < .001) and objective assessments (SPPB scores: WSMDs 4.6 ± 3.3, compared to WAs at 5.9 ± 2.6 and Mixed at 5.0 ± 2.6, *p* < .001), as well as poorer cognitive function (Clock drawing test scores: WSMDs 3.3 ± 1.4, compared to WAs at 3.6 ± 1.1 and Mixed at 3.4 ± 1.2, *p* < .001). Both WSMDs and mixed device users were more likely to have lower grip strength (average: WSMDs 22.2 ± 10.1 lbs. and Mixed 22.4 ± 8.6 lbs., compared to WAs at 23.9 ± 8.9 lbs., *p* < .001; maximum: WSMDs 23.3 ± 10.7 lbs. and Mixed 23.4 ± 8.7 lbs., compared to WAs at 24.9 ± 9.1 lbs., *p* < .001). Notably, mixed device users were the most easily exhausted group, represented by the highest prevalence of presenting exhaustion (20.4%, compared to WAs at 18.8% and WSMDs at 16.6%, *p* < .001) and exhaustion limiting activities (Mixed 41.6%, compared to WAs at 34.5% and WSMDs at 41.5%, *p* < .001).

Significant differences in *accommodations* were observed among the groups, especially in the need for personal assistance with mobility. WSMDs users showed a higher dependence (0.4 ± 0.7) at the baseline, followed by mixed device users (0.2 ± 0.5), in comparison to WAs users (0.1 ± 0.3, *p* < .001).

Table 2 presents an analysis of the baseline characteristics of community-dwelling older mobility device users, focusing on the *number of mobility device combination changes* they experienced during their later device use. Regarding *personal factors*, device users with changes in mobility device combinations were older (2+ Changes 80.5 ± 7.0 years and 1 Change 81.6 ± 7.2 years, compared to No Change at 79.9 ± 7.2 years, *p* < .001), and more likely to be widowed (2+ Changes 44.0% and 1 Change 41.8%, compared to No Change at 36.6%, *p* < .001).

In terms of *health conditions*, device users with two or more changes were in poorer general health (3.1 ± 1.0, compared to No Change at 3.0 ± 1.0 and 1 Change at 3.0 ± 1.0, *p* < .05). Generally, users who changed their mobility device combinations reported falls more frequently (multiple falls: 2+ Changes 25.0% and 1 Change 24.2%, compared to No Change at 14.9%, *p* < .001) and were more likely to report fear of falling that limited their activities (2+ Changes 17.8% and 1 Change 14.9%, compared to No Change at 10.4%, *p* < .001). Breathing problems that limited their activities were also more frequent (2+ Changes 18.7% and 1 Change 14.5%, compared to No Change at 12.2%, *p* < .05).

The body *impairments* and functional *capacity* of device users who changed their mobility device combinations also showed increased risks for decline. These users exhibited more impairments in their lower limbs (2+ Changes 32.5% and 1 Change 30.9%, compared to No Change at 25.4%, *p* < .05) and balance (2+ Changes 27.9% and 1 Change 23.5%, compared to No Change at 17.1%, *p* < .001) that limited their activities. They also showed worse mobility capacity in both subjective (Self-reported mobility impairments: 2+ Changes 1.7 ± 1.5 and 1 Change 1.3 ± 1.5, compared to No Change at 1.1 ± 1.4, *p* < .001) and objective assessments (SPPB scores: 2+ Changes 5.0 ± 2.4 and 1 Change 5.1 ± 2.5, compared to No Change at 5.7 ± 2.8, *p* < .001), as well as poorer cognitive function (Clock drawing test scores: 2+ Changes 3.5 ± 1.1 and 1 Change 3.4 ± 1.2, compared to No Change at 3.6 ± 1.1, *p* < .01). Mobility device users with two or more changes demonstrated significantly weaker grip strength (average: 21.8 ± 8.0 lbs., compared to No Change at 23.7 ± 8.9 lbs. and 1 Change at 23.0 ± 9.5 lbs., *p* < .01; maximum: 22.8 ± 8.2 lbs., compared to No Change at 24.7 ± 9.0 lbs. and 1 Change at 24.0 ± 9.7 lbs., *p* < .01). However, no significant differences were observed in the domain of *accommodations*.

### 4.3 | Older mobility device users’ experience in community settings

The experience of older mobility device users in community settings was closely related to two primary aspects: the *types of mobility devices* and the *number of mobility device combination changes*. Table 3 illustrates the older mobility device users’ experience by *types of mobility devices*, revealing particularly noteworthy findings regarding mixed device users. These users had a longer duration of mobility device use (2.9 ± 1.9 years, compared to WAs at 1.9 ± 1.5 years and WSMDs at 1.3 ± 0.7 years, *p* < .001), and also changed their device combinations more frequently (1.2 ± 1.2 times, compared to WAs at 0.2 ± 0.6 times and WSMDs at 0.0 ± 0.2 times, *p* < .001). Mixed device users were more dependent on mobility devices outdoors (3.0 ± 1.1, compared to WAs at 2.8 ± 1.2 and WSMDs at 2.6 ± 1.7, *p* < .001) and they also reported higher difficulty levels when using mobility devices outdoors (1.5 ± 0.7, compared to WAs at 1.3 ± 0.5 and WSMDs at 1.3 ± 0.6, *p* < .001). When moving indoors, mixed device users more frequently relied on holding onto walls or furniture (2.5 ± 1.0, compared to WAs at 2.3 ± 0.9 and WSMDs at 2.2 ± 1.0, *p* < .001). Both WSMDs and mixed device users showed a dependency on indoor mobility device use (WSMDs 2.9 ± 1.9 and Mixed 2.7 ± 1.1, compared to WAs at 2.2 ± 1.1, *p* < .001) and similar higher difficulty levels when using them indoors (WSMDs 1.5 ± 0.7 and Mixed 1.5 ± 0.6, compared to WAs at 1.3 ± 0.5, *p* < .001). These groups also incurred higher costs on assistive products (WSMDs 1.9 ± 0.8 and Mixed 1.9 ± 0.8, compared to WAs at 1.8 ± 0.8, *p* < .05).

Table 4, stratified by the *number of mobility device combination changes*, highlights a generally worse user experience among those who changed their device combinations. These users had a longer duration of mobility device use (2+ Changes 4.9 ± 1.5 years and 1 Change 3.0 ± 1.4 years, compared to No Change at 1.5 ± 1.0 years, *p* < .001), predominantly among mixed device users (2+ Changes 77.3% and 1 Change 57.5%, compared to No Change at 14.1%, *p* < .001). They also reported a higher frequency of using devices both indoors (2+ Changes 2.6 ± 0.9 and 1 Change 2.6 ± 1.0, compared to No Change at 2.2 ± 1.2, *p* < .001) and outdoors (2+ Changes 3.3 ± 0.8 and 1 Change 3.2 ± 1.1, compared to No Change at 2.7 ± 1.3, *p* < .001). Additionally, these users were more dependent on holding onto walls and furniture when moving indoors (2+ Changes 2.7 ± 0.9 and 1 Change 2.6 ± 1.0, compared to No Change at 2.2 ± 0.9, *p* < .001). They experienced higher levels of difficulty when using mobility devices both indoors (2+ Changes 1.6 ± 0.5 and 1 Change 1.5 ± 0.5, compared to No Change at 1.4 ± 0.6, *p* < .001) and outdoors (2+ Changes 1.6 ± 0.6 and 1 Change 1.5 ± 0.6, compared to No Change at 1.3 ± 0.5, *p* < .001).

## 5 | DISCUSSION

To the best of our knowledge, this study is the first to provide longitudinal observations on the use of a comprehensive range of mobility devices in community settings among a nationally representative sample of older adults. Our findings reveal that the trajectories of incident mobility device use among community-dwelling older adults are dynamic, typically involving relatively short durations of use, a variety of combinations across different types of mobility devices, and frequent changes in these combinations over time. While previous studies have observed frequent changes in mobility device usage status,^11,25,26^ types of mobility devices used,^27,28^ and combination usage^85^ separately, this study is the first to summarize all these dynamic patterns in one cohort. This finding suggests that older adults have dynamic needs for mobility devices, potentially due to changes in other *accommodation* factors, such as environmental accessibility and personal assistance, as described in the NHATS late-life disability framework.^117^ Future research on mobility devices should pay particular attention to these dynamic usage patterns and further explore the underlying reasons for these changes.

The majority of older mobility device users used walking aids, but there was a noticeable shift towards the use of wheeled and seated mobility devices or mixed device use for increased support. This trend suggests a potential emerging mobility disability and an increasing dependence on mobility devices among community-dwelling older adults over time, aligning with findings from previous studies globally.^11,25,26^ Given the projected increase in the aging population globally,^131^ we anticipate a growing demand for mobility devices, especially wheeled and seated ones, in the foreseeable future. This trend will pose both opportunities and challenges for mobility device service delivery, as well as for the implementation of environmental adaptations and the availability of personal assistance to facilitate older people’s use of mobility devices in community settings.

Compared to other types of mobility device users, older adults who used wheeled and seated mobility devices were found to be in the poorest health status, with more chronic conditions and lower functional capacities at baseline. They also showed higher dependence on others, either living with others or needing mobility assistance indoors and outdoors, before starting to use wheelchairs or scooters. Older users of wheeled and seated mobility devices were more likely to rely on others for activities of daily livings (ADLs),^132^ and this study revealed their pre-existing reliance on personal assistance even before starting to use any devices. Current research has shown that the use of mobility devices can substitute for personal assistance for mobility;^62^ however, future research should further explore whether they may also alleviate older users’ reliance on personal assistance for other activities and participations.

Regarding user experience, the mixed device users reported the worst experience. During their prolonged use of devices, they changed the device combination frequently with higher overall costs, exhibited high reliance on mobility devices for indoor and outdoor mobility, and faced higher difficulties in using these devices. This pattern of mixed device use has been reported in previous research,^85^ which often involves adopting different types of devices to cope with different environments. However, frequent changes between walking aids and wheeled and seated mobility devices, which involve different ambulation mechanisms, may increase the risk of falls,^133–135^ especially since these older mobility device users are already at a high risk of falls at baseline. Users of walking aids were particularly noted for severe pain and heavier body weight, both of which are associated with osteoarthritis, the most prevalent form of arthritis affecting one in three older adults worldwide.^136^ Walking aids are recommended in guidelines for managing such conditions.^137,138^ This finding demonstrates the adaptive behaviors of older adults in managing arthritis-related symptoms through the use of walking aids.

There were significant differences observed between older users who did not change their mobility device combinations and those who did, sometimes showing a dose-responsive pattern. Overall, older adults who changed their mobility device combination were found to be in a more vulnerable state: they were typically older and widowed, with poorer health conditions and functional capacities. Their user experience with mobility devices was also less favorable, characterized by prolonged use, higher reliance on mobility devices both indoors and outdoors, and greater difficulties in using them. Previous research has noted changing patterns of mobility device use, especially following hospital discharge,^27,28^ which reflected evolving or unmet needs among users. The concern, however, is not merely the related costs of frequent device changes but also whether these decisions are made with professional guidance and appropriate assessment, rather than solely by the users themselves, as self-determined device changes may introduce risks during use.^28^ Future research is needed to investigate the rationales behind these frequent changing patterns in mobility device use and to assess their potential impacts on the safe use of mobility devices among older people.

## 6 | POLICY IMPLICATIONS

The observed patterns of mobility device use among community-dwelling older adults, characterized by short durations, combination use, and frequent changes, may offer valuable insights for policymakers, facilitating their informed decisions on resource allocation and the development of service delivery frameworks. For example, given the short duration of mobility device use, a rental program could be more appropriate than one-time purchases for older people in communities. For those frequently changing their mobility device combinations, a recycling platform could effectively manage their abandoned devices. However, existing rental and recycling programs for mobility devices face significant barriers, such as challenges in navigating the procurement process, inadequate insurance coverage, and a lack of awareness of insurance benefits.^139^ Enhancing support for these programs could involve leveraging additional resources from nongovernmental organizations and professional associations like the Association of Assistive Technology Act Programs (ATAP) and the Rehabilitation Engineering and Assistive Technology Society of North America (RESNA). This approach would not only benefit device recipients but also significantly contribute to environmental conservation.^140^

Access to mobility devices in the United States is primarily covered by Medicare, Medicaid, and private insurance, as mandated by the Affordable Care Act.^141^ For older Medicare beneficiaries, the focus of this study, mobility devices are typically acquired through Medicare and classified as durable medical equipment (DME).^142^ This policy requires that the need for DME be reviewed annually by a healthcare professional, such as a physician, nurse practitioner, clinical nurse specialist, or physician assistant. While this policy accommodates frequent changes in the types of mobility devices required, it does not support the possession of multiple devices; for instance, a backup mobility device is often not covered by insurance.^139^ A more flexible policy that accommodates not only frequent changes but also the possession of multiple mobility devices, or even exchanges between types of mobility devices, may better suit the needs of Medicare beneficiaries. Additionally, developing integrated mobility devices that combine features of walking aids and wheeled and seated ambulation, which could better meet the needs and cost-sensitive preferences of older users.^36^

This study also identified subgroups of older mobility device users who are at higher vulnerability in communities, specifically those using wheeled and seated mobility devices and those frequently changing their device combinations. Tailored policies favoring these groups could significantly enhance health equity for the U.S. older population. Practical suggestions may involve connecting social determinants of health (SDOH) programs at the mobility device service delivery sites. Additionally, certain subgroups were found to need more support during the service delivery process to enhance their user experience, including those using mixed devices and those frequently changing their device combinations. Suggestions for stakeholders in mobility device service delivery extend beyond mobility device-related services to include other *accommodation* factors that impact older mobility device users’ experiences in communities, such as the implementation of environmental modifications and the provision of personal assistance.

## 7 | LIMITATIONS

This study was conducted following the NHATS late-life disability framework.^117^ While this framework informed the data collection of the NHATS cohort, certain aspects influencing mobility device use were not fully addressed, such as the use of assistant animals and the users’ attitudes towards using mobility devices. Moreover, the cohort design makes it challenging to differentiate the actual end of mobility device use from loss to follow-up, moving out of community settings, or death, possibly leading to underestimates of use duration. Our analysis is descriptive, exploring the patterns of mobility device use, their influencing factors, and user experience among community-dwelling older adults. This approach limits our ability to establish causal relationships between influencing factors and the patterns of mobility device use.

The measurement of mobility device use was assessed annually, focusing on the using pattern in the past month before the interview. This approach may have overlooked changes that occurred between these assessment periods. Additionally, as patterns and user experiences of using mobility devices can vary with seasons,^143^ the timing of in-home interviews could introduce potential biases. Furthermore, the records were limited to identifying the types of mobility devices used, so changes within the same category, such as replacing an old cane with a new one, could not be captured.

Another limitation is the time-varying nature of user experience, which was not analyzed in a robust time-varying manner due to a rapid decrease in sample size over time. Furthermore, the nuances of user experiences, often intricate and subjective, cannot be fully captured by quantitative questionnaires. Therefore, qualitative research involving long-term follow-up is necessary to gain a deeper understanding of the user experiences regarding mobility devices among community-dwelling older adults. This is particularly important for those using mixed devices and those frequently changing device combinations.

## 8 | CONCLUSION

This longitudinal descriptive study identified patterns of mobility device use among a nationally representative sample of community-dwelling older adults in the United States, characterized by short durations, combination use, and frequent changes, with notable trends showing an increasing dependency from walking aids to mixed and wheeled and seated mobility devices. The study advocates for the implementation of rental and recycling programs, and the involvement of nongovernmental organizations and professional associations, along with more flexible policies that adapt to the possession of multiple devices and the exchange among different types of mobility devices. The study also identified vulnerable subgroups of older mobility device user, particularly those using wheeled and seated mobility devices and those frequently changing their device combinations, emphasizing the potential for addressing social determinants of health at mobility device service delivery sites. To enhance the user experience for those with mixed devices and frequent device changes, mobility device service delivery should extend to cover a broader range of accommodation factors, such as the implementation of environmental modifications and the provision of personal assistance.

## Figures and Tables

**TABLE 1:** Baseline characteristics of mobility device users, by types of mobility devices.

	Total N = 2,943	WAs N = 1,877	Mixed N = 873	WSMDs N = 193	P-value

Age, Mean (SD)	80.3 (7.2)	79.8 (7.0)	81.1 (7.4)	81.3 (8.0)	<0.001
Female, N (%)	1,697 (57.7%)	1,084 (57.8%)	503 (57.6%)	110 (57.0%)	0.98
Race and Ethnicity, N (%)					0.82
White, non-Hispanic	2,066 (70.2%)	1,300 (69.3%)	634 (72.6%)	132 (68.4%)	
Black, non-Hispanic	590 (20.0%)	393 (20.9%)	157 (18.0%)	40 (20.7%)	
Other, non-Hispanic	81 (2.8%)	54 (2.9%)	20 (2.3%)	7 (3.6%)	
Hispanic	167 (5.7%)	104 (5.5%)	51 (5.8%)	12 (6.2%)	
Multiracial	1 (0.0%)	1 (0.1%)	0 (0.0%)	0 (0.0%)	
Education Level, N (%)					0.064
Less than high school	761 (25.9%)	473 (25.2%)	235 (26.9%)	53 (27.5%)	
High school diploma	779 (26.5%)	485 (25.8%)	255 (29.2%)	39 (20.2%)	
Some college and Bachelor’s	1,074 (36.5%)	694 (37.0%)	297 (34.0%)	83 (43.0%)	
Graduate education	294 (10.0%)	198 (10.5%)	79 (9.0%)	17 (8.8%)	
Marital Status, N (%)					<0.05
Married or with a partner	1,356 (46.1%)	868 (46.2%)	400 (45.8%)	88 (45.6%)	
Single	455 (15.5%)	314 (16.7%)	109 (12.5%)	32 (16.6%)	
Widowed	1,131 (38.4%)	695 (37.0%)	363 (41.6%)	73 (37.8%)	
Household Income, N (%)					0.98
Poorest 0–25%	459 (15.6%)	292 (15.6%)	141 (16.2%)	26 (13.5%)	
25%–50%	516 (17.5%)	326 (17.4%)	157 (18.0%)	33 (17.1%)	
50%–75%	425 (14.4%)	267 (14.2%)	129 (14.8%)	29 (15.0%)	
75%–100% Richest	359 (12.2%)	231 (12.3%)	106 (12.1%)	22 (11.4%)	
Number of Children, N (%)					0.23
No children	243 (8.3%)	169 (9.0%)	58 (6.6%)	16 (8.3%)	
Single child	343 (11.7%)	225 (12.0%)	99 (11.3%)	19 (9.8%)	
Two or more children	2,357 (80.1%)	1,483 (79.0%)	716 (82.0%)	158 (81.9%)	
Metropolitan Status, N (%)	2,353 (80.0%)	1,502 (80.0%)	688 (78.8%)	163 (84.5%)	0.21
Community Environment, Mean (SD)	7.0 (1.9)	7.0 (1.9)	7.1 (1.9)	7.2 (1.7)	0.31
Housing Type, N (%)					<0.001
Free-standing single house	2,190 (74.4%)	1,379 (73.5%)	661 (75.7%)	150 (77.7%)	
House attached to others	252 (8.6%)	167 (8.9%)	69 (7.9%)	16 (8.3%)	
Mobile home/trailer	158 (5.4%)	83 (4.4%)	66 (7.6%)	9 (4.7%)	
Multi-unit building	332 (11.3%)	238 (12.7%)	77 (8.8%)	17 (8.8%)	
Other	11 (0.4%)	10 (0.5%)	0 (0.0%)	1 (0.5%)	
Vehicle Ownership, N (%)	2,309 (80.6%)	1,481 (81.4%)	685 (80.2%)	143 (75.7%)	0.15
Living Arrangement, N (%)					<0.001
Living alone	1,019 (34.6%)	685 (36.5%)	284 (32.5%)	50 (25.9%)	
Living with a spouse/partner	1,067 (36.3%)	697 (37.1%)	305 (34.9%)	65 (33.7%)	
Living with a spouse/partner and others	265 (9.0%)	153 (8.2%)	91 (10.4%)	21 (10.9%)	
Living with others	591 (20.1%)	341 (18.2%)	193 (22.1%)	57 (29.5%)	
General Health, Mean (SD)	3.0 (1.0)	2.9 (1.0)	3.1 (1.0)	3.2 (1.1)	<0.001
Chronic Diseases, Mean (SD)	2.0 (1.2)	1.9 (1.2)	2.0 (1.3)	2.2 (1.2)	<0.01
Surgery History, Mean (SD)	0.6 (0.8)	0.6 (0.8)	0.6 (0.8)	0.6 (0.9)	0.91
Hip Fracture History, N (%)	166 (5.6%)	108 (5.8%)	51 (5.8%)	7 (3.6%)	0.45
Other Fracture History, N (%)	742 (25.2%)	477 (25.4%)	223 (25.6%)	42 (21.8%)	0.52
Fall History, N (%)					<0.001
No fall	1,858 (63.1%)	1,208 (64.4%)	516 (59.1%)	134 (69.4%)	
One fall	723 (24.6%)	466 (24.8%)	221 (25.3%)	36 (18.7%)	
Multiple falls	357 (12.1%)	201 (10.7%)	135 (15.5%)	21 (10.9%)	
Fear of Falling, N (%)					<0.001
No fear of falling	1858 (63.1%)	1,344 (65.7%)	330 (60.0%)	184 (52.9%)	
Fear of falling presents	723 (24.6%)	486 (23.8%)	138 (25.1%)	99 (28.4%)	
Fear of falling limits activities	357 (12.1%)	213 (10.4%)	82 (14.9%)	62 (17.8%)	
Pain, N (%)					<0.05
No pain	1,186 (40.3%)	719 (38.3%)	374 (42.8%)	93 (48.2%)	
Bothered by pain	873 (29.7%)	588 (31.3%)	239 (27.4%)	46 (23.8%)	
Pain limits activities	880 (29.9%)	568 (30.3%)	259 (29.7%)	53 (27.5%)	
Breathing Problems, N (%)					<0.001
No breathing problems	2,165 (73.6%)	1,418 (75.5%)	611 (70.0%)	136 (70.5%)	
Breathing problems present	380 (12.9%)	242 (12.9%)	118 (13.5%)	20 (10.4%)	
Problems limit activities	395 (13.4%)	215 (11.5%)	144 (16.5%)	36 (18.7%)	
BMI, N (%)					<0.001
Underweight (< 18.5 kg/m^2^)	83 (2.8%)	36 (1.9%)	36 (4.1%)	11 (5.7%)	
Normal (18.5–24.9 kg/m^2^)	934 (31.7%)	561 (29.9%)	292 (33.4%)	81 (42.0%)	
Overweight (25.0–29.9 kg/m^2^)	993 (33.7%)	673 (35.9%)	265 (30.4%)	55 (28.5%)	
Obesity (≥30 kg/m^2^)	933 (31.7%)	607 (32.3%)	280 (32.1%)	46 (23.8%)	
PHQ-2 Scores, Mean (SD)	1.1 (1.4)	1.0 (1.3)	1.2 (1.5)	1.2 (1.5)	<0.05
GAD-2 Scores, Mean (SD)	0.9 (1.4)	0.9 (1.3)	1.1 (1.5)	1.1 (1.6)	<0.001
Vision Impairments, Mean (SD)	0.2 (0.5)	0.2 (0.4)	0.3 (0.5)	0.4 (0.6)	<0.001
Lower Limb Impairments, N (%)					0.19
No impairments	1,712 (58.2%)	1,102 (58.7%)	489 (56.0%)	121 (62.7%)	
Impairments present	423 (14.4%)	284 (15.1%)	117 (13.4%)	22 (11.4%)	
Impairments limit activities	803 (27.3%)	488 (26.0%)	265 (30.4%)	50 (25.9%)	
Low Energy, N (%)					<0.001
No exhaustion	1,281 (43.5%)	873 (46.5%)	330 (37.8%)	78 (40.4%)	
Exhaustion presents	562 (19.1%)	352 (18.8%)	178 (20.4%)	32 (16.6%)	
Exhaustion limits activities	1,090 (37.0%)	647 (34.5%)	363 (41.6%)	80 (41.5%)	
Balance impairments, N (%)					<0.001
No balance problems	1,703 (57.9%)	1,133 (60.4%)	463 (53.0%)	107 (55.4%)	
Balance problems present	662 (22.5%)	415 (22.1%)	211 (24.2%)	36 (18.7%)	
Problems limit activities	575 (19.5%)	328 (17.5%)	198 (22.7%)	49 (25.4%)	
Mobility Impairments, Mean (SD)	1.2 (1.5)	1.1 (1.4)	1.5 (1.5)	1.7 (1.7)	<0.001
SPPB Scores, Mean (SD)	5.5 (2.7)	5.9 (2.6)	5.0 (2.6)	4.6 (3.3)	<0.001
Average Grip Strength, kg.	23.4 (8.9)	23.9 (8.9)	22.4 (8.6)	22.2 (10.1)	<0.001
Maximum Grip Strength, kg.	24.3 (9.1)	24.9 (9.1)	23.4 (8.7)	23.3 (10.7)	<0.001
Clock Drawing Test Scores, Mean (SD)	3.5 (1.1)	3.6 (1.1)	3.4 (1.2)	3.3 (1.4)	<0.001
Stairs at Entrance, N (%)	2,074 (70.6%)	1,313 (70.1%)	621 (71.2%)	140 (72.5%)	0.68
Stairs in Home, N (%)	1,299 (47.2%)	842 (47.7%)	370 (45.7%)	87 (48.1%)	0.62
Bathroom Modifications, Mean (SD)	2.1 (1.0)	2.0 (1.0)	2.1 (1.0)	2.1 (1.0)	0.11
Mobility Assistance, Mean (SD)	0.1 (0.4)	0.1 (0.3)	0.2 (0.5)	0.4 (0.7)	<0.001

*Note*: WAs, walking aids; WSMDs, wheeled and seated mobility devices; Mixed, concurrent use of both WAs and WSMDs; SD, standard deviation; BMI, body mass index; PHQ-2, Patient Health Questionnaire-2 items; GAD-2, Generalized Anxiety Disorder-2 items; SPPB, Short Physical Performance Battery.

**TABLE 2:** Baseline characteristics of mobility device users, by number of mobility device combination changes.

	Total N = 2,943	No Change N = 2,045	1 Change N = 550	2+ Changes N = 348	*p*-value

Age, Mean (SD)	80.3 (7.2)	79.9 (7.2)	81.6 (7.2)	80.5 (7.0)	<0.001
Female, N (%)	1,697 (57.7%)	1,157 (56.6%)	319 (58.0%)	221 (63.5%)	0.053
Race and Ethnicity, N (%)					0.088
White, non-Hispanic	2,066 (70.2%)	1,431 (70.0%)	395 (71.8%)	240 (69.0%)	
Black, non-Hispanic	590 (20.0%)	401 (19.6%)	106 (19.3%)	83 (23.9%)	
Other, non-Hispanic	81 (2.8%)	62 (3.0%)	15 (2.7%)	4 (1.1%)	
Hispanic	167 (5.7%)	123 (6.0%)	27 (4.9%)	17 (4.9%)	
Multiracial	1 (0.0%)	0 (0.0%)	0 (0.0%)	1 (0.3%)	
Education Level, N (%)					0.61
Less than high school	761 (25.9%)	521 (25.5%)	143 (26.0%)	97 (27.9%)	
High school diploma	779 (26.5%)	532 (26.0%)	148 (26.9%)	99 (28.4%)	
Some college and Bachelor’s	1,074 (36.5%)	752 (36.8%)	201 (36.5%)	121 (34.8%)	
Graduate education	294 (10.0%)	211 (10.3%)	53 (9.6%)	30 (8.6%)	
Marital Status, N (%)					<0.001
Married or with a partner	1,356 (46.1%)	945 (46.2%)	251 (45.6%)	160 (46.0%)	
Single	455 (15.5%)	352 (17.2%)	69 (12.5%)	34 (9.8%)	
Widowed	1,131 (38.4%)	748 (36.6%)	230 (41.8%)	153 (44.0%)	
Household Income, N (%)					0.44
Poorest 0–25%	459 (15.6%)	308 (15.1%)	91 (16.5%)	60 (17.2%)	
25%–50%	516 (17.5%)	361 (17.7%)	99 (18.0%)	56 (16.1%)	
50%–75%	425 (14.4%)	306 (15.0%)	72 (13.1%)	47 (13.5%)	
75%–100% Richest	359 (12.2%)	251 (12.3%)	56 (10.2%)	52 (14.9%)	
Number of Children, N (%)					0.29
No children	243 (8.3%)	182 (8.9%)	39 (7.1%)	22 (6.3%)	
Single child	343 (11.7%)	228 (11.1%)	70 (12.7%)	45 (12.9%)	
Two or more children	2,357 (80.1%)	1,635 (80.0%)	441 (80.2%)	281 (80.7%)	
Metropolitan Status, N (%)	2,353 (80.0%)	1,644 (80.4%)	435 (79.1%)	274 (78.7%)	0.66
Community Environment, Mean (SD)	7.0 (1.9)	7.0 (1.9)	7.1 (1.8)	7.0 (1.8)	0.85
Housing Type, N (%)					0.44
Free-standing single house	2,190 (74.4%)	1,513 (74.0%)	419 (76.2%)	258 (74.1%)	
House attached to others	252 (8.6%)	172 (8.4%)	50 (9.1%)	30 (8.6%)	
Mobile home/trailer	158 (5.4%)	108 (5.3%)	30 (5.5%)	20 (5.7%)	
Multi-unit building	332 (11.3%)	241 (11.8%)	51 (9.3%)	40 (11.5%)	
Other	11 (0.4%)	11 (0.5%)	0 (0.0%)	0 (0.0%)	
Vehicle Ownership, N (%)	2,309 (80.6%)	1,598 (80.5%)	442 (81.9%)	269 (79.6%)	0.68
Living Arrangement, N (%)					0.56
Living alone	1,019 (34.6%)	710 (34.7%)	186 (33.8%)	123 (35.3%)	
Living with a spouse/partner	1,067 (36.3%)	757 (37.0%)	191 (34.7%)	119 (34.2%)	
Living with a spouse/partner and others	265 (9.0%)	169 (8.3%)	56 (10.2%)	40 (11.5%)	
Living with others	591 (20.1%)	408 (20.0%)	117 (21.3%)	66 (19.0%)	
General Health, Mean (SD)	3.0 (1.0)	3.0 (1.0)	3.0 (1.0)	3.1 (1.0)	<0.05
Chronic Diseases, Mean (SD)	2.0 (1.2)	2.0 (1.2)	2.0 (1.3)	1.9 (1.2)	0.37
Surgery History, Mean (SD)	0.6 (0.8)	0.6 (0.8)	0.7 (0.8)	0.6 (0.8)	0.31
Hip Fracture History, N (%)	166 (5.6%)	109 (5.3%)	34 (6.2%)	23 (6.6%)	0.53
Other Fracture History, N (%)	742 (25.2%)	504 (24.7%)	134 (24.4%)	104 (29.9%)	0.10
Fall History, N (%)					<0.001
No fall	1,826 (62.0%)	1,325 (64.8%)	304 (55.3%)	197 (56.6%)	
One fall	586 (19.9%)	414 (20.2%)	110 (20.0%)	62 (17.8%)	
Multiple falls	524 (17.8%)	304 (14.9%)	133 (24.2%)	87 (25.0%)	
Fear of Falling, N (%)					<0.001
No fear of falling	1,858 (63.1%)	1344 (65.7%)	330 (60.0%)	184 (52.9%)	
Fear of falling presents	723 (24.6%)	486 (23.8%)	138 (25.1%)	99 (28.4%)	
**Fear** of falling limits activities	357 (12.1%)	213 (10.4%)	82 (14.9%)	62 (17.8%)	
Pain, N (%)					0.21
No pain	1,186 (40.3%)	845 (41.3%)	224 (40.7%)	117 (33.6%)	
Bothered by pain	873 (29.7%)	602 (29.4%)	158 (28.7%)	113 (32.5%)	
Pain limits activities	880 (29.9%)	595 (29.1%)	167 (30.4%)	118 (33.9%)	
Breathing Problems, N (%)					<0.05
No breathing problems	2165 (73.6%)	1,522 (74.4%)	404 (73.5%)	239 (68.7%)	
Breathing problems present	380 (12.9%)	270 (13.2%)	66 (12.0%)	44 (12.6%)	
Problems limit activities	395 (13.4%)	250 (12.2%)	80 (14.5%)	65 (18.7%)	
BMI, N (%)					0.11
Underweight (<18.5 kg/m^2^)	83 (2.8%)	61 (3.0%)	13 (2.4%)	9 (2.6%)	
Normal (18.5–24.9 kg/m^2^)	934 (31.7%)	660 (32.3%)	174 (31.6%)	100 (28.7%)	
Overweight (25.0–29.9 kg/m^2^)	993 (33.7%)	706 (34.5%)	182 (33.1%)	105 (30.2%)	
Obesity (≥30 kg/m^2^)	933 (31.7%)	618 (30.2%)	181 (32.9%)	134 (38.5%)	
PHQ-2 Scores, Mean (SD)	1.1 (1.4)	1.1 (1.4)	1.1 (1.5)	1.1 (1.5)	0.49
GAD-2 Scores, Mean (SD)	0.9 (1.4)	0.9 (1.4)	1.0 (1.4)	1.0 (1.5)	0.26
Vision Impairments, Mean (SD)	0.2 (0.5)	0.2 (0.5)	0.2 (0.5)	0.2 (0.4)	0.30
Lower Limb Impairments, N (%)					<0.05
No impairments	1,712 (58.2%)	1,232 (60.2%)	300 (54.5%)	180 (51.7%)	
Impairments present	423 (14.4%)	289 (14.1%)	79 (14.4%)	55 (15.8%)	
Impairments limit activities	803 (27.3%)	520 (25.4%)	170 (30.9%)	113 (32.5%)	
Low Energy, N (%)					0.15
No exhaustion	1,281 (43.5%)	927 (45.3%)	219 (39.8%)	135 (38.8%)	
Exhaustion presents	562 (19.1%)	377 (18.4%)	116 (21.1%)	69 (19.8%)	
Exhaustion limits activities	1,090 (37.0%)	734 (35.9%)	213 (38.7%)	143 (41.1%)	
Balance impairments, N (%)					<0.001
No balance problems	1,703 (57.9%)	1,249 (61.1%)	303 (55.1%)	151 (43.4%)	
Balance problems present	662 (22.5%)	445 (21.8%)	118 (21.5%)	99 (28.4%)	
Problems limit activities	575 (19.5%)	349 (17.1%)	129 (23.5%)	97 (27.9%)	
Mobility Impairments, Mean (SD)	1.2 (1.5)	1.1 (1.4)	1.3 (1.5)	1.7 (1.5)	<0.001
SPPB Scores, Mean (SD)	5.5 (2.7)	5.7 (2.8)	5.1 (2.5)	5.0 (2.4)	<0.001
Average Grip Strength, kg.	23.4 (8.9)	23.7 (8.9)	23.0 (9.5)	21.8 (8.0)	<0.01
Maximum Grip Strength, kg.	24.3 (9.1)	24.7 (9.0)	24.0 (9.7)	22.8 (8.2)	<0.01
Clock Drawing Test Scores, Mean (SD)	3.5 (1.1)	3.6 (1.1)	3.4 (1.2)	3.5 (1.1)	<0.01
Stairs at Entrance, N (%)	2,074 (70.6%)	1448 (70.9%)	380 (69.2%)	246 (70.7%)	0.74
Stairs in Home, N (%)	1,299 (47.2%)	910 (47.3%)	234 (46.0%)	155 (48.1%)	0.81
Bathroom Modifications, Mean (SD)	2.1 (1.0)	2.1 (1.0)	2.1 (1.0)	2.1 (1.1)	0.24
Mobility Assistance, Mean (SD)	0.1 (0.4)	0.1 (0.4)	0.1 (0.4)	0.2 (0.5)	0.12

*Note*: WAs, walking aids; WSMDs, wheeled and seated mobility devices; Mixed, concurrent use of both WAs and WSMDs; SD, standard deviation; BMI, body mass index; PHQ-2, Patient Health Questionnaire-2 items; GAD-2, Generalized Anxiety Disorder-2 items; SPPB, Short Physical Performance Battery.

**TABLE 3:** Mobility device use-related characteristics, by types of mobility devices.

	Total N = 2,943	WAs N = 1,877	Mixed N = 873	WSMDs N = 193	*p*-value

Duration of incident mobility device use, years	2.2 (1.6)	1.9 (1.5)	2.9 (1.9)	1.3 (0.7)	<0.001
Number of Mobility Device Combination Changes, Mean (SD)	0.5 (0.9)	0.2 (0.6)	1.2 (1.2)	0.0 (0.2)	<0.001
Frequency of using mobility device indoors, Mean (SD)	2.4 (1.1)	2.2 (1.1)	2.7 (1.1)	2.9 (1.9)	<0.001
Frequency of using mobility device outdoors, Mean (SD)	2.9 (1.2)	2.8 (1.2)	3.0 (1.1)	2.6 (1.7)	<0.001
Frequency of holding onto walls/furniture when moving indoors, Mean (SD)	2.3 (1.0)	2.3 (0.9)	2.5 (1.0)	2.2 (1.0)	<0.001
Difficulty of using mobility device indoors, Mean (SD)	1.4 (0.6)	1.3 (0.5)	1.5 (0.6)	1.5 (0.7)	<0.001
Difficulty of using mobility device outdoors, Mean (SD)	1.4 (0.6)	1.3 (0.5)	1.5 (0.7)	1.3 (0.6)	<0.001
Costs on assistive products, Mean (SD)	1.8 (0.8)	1.8 (0.8)	1.9 (0.8)	1.9 (0.8)	<0.05

*Note*: WAs, walking aids; WSMDs, wheeled and seated mobility devices; Mixed, concurrent use of both WAs and WSMDs.

**TABLE 4:** Mobility device use-related characteristics, by number of mobility device combination changes.

	Total N = 2,943	No Change N = 2,045	1 Change N = 550	2+ Changes N = 348	*P*-value

Duration of incident mobility device use, years	2.2 (1.6)	1.5 (1.0)	3.0 (1.4)	4.9 (1.5)	<0.001
Types of mobility devices, N (%)					<0.001
WAs	1,877 (63.8%)	1571 (76.8%)	227 (41.3%)	79 (22.7%)	
Mixed	873 (29.7%)	288 (14.1%)	316 (57.5%)	269 (77.3%)	
WSMDs	193 (6.6%)	186 (9.1%)	7 (1.3%)	0 (0.0%)	
Frequency of using mobility device indoors, Mean (SD)	2.4 (1.1)	2.2 (1.2)	2.6 (1.0)	2.6 (0.9)	<0.001
Frequency of using mobility device outdoors, Mean (SD)	2.9 (1.2)	2.7 (1.3)	3.2 (1.1)	3.3 (0.8)	<0.001
Frequency of holding onto walls/furniture when moving indoors, Mean (SD)	2.3 (1.0)	2.2 (0.9)	2.6 (1.0)	2.7 (0.9)	<0.001
Difficulty of using mobility device indoors, Mean (SD)	1.4 (0.6)	1.4 (0.6)	1.5 (0.5)	1.6 (0.5)	<0.001
Difficulty of using mobility device outdoors, Mean (SD)	1.4 (0.6)	1.3 (0.5)	1.5 (0.6)	1.6 (0.6)	<0.001
Costs on assistive products, Mean (SD)	1.8 (0.8)	1.8 (0.8)	1.9 (0.8)	1.8 (0.7)	0.93

*Note*: WAs, walking aids; WSMDs, wheeled and seated mobility devices; Mixed, concurrent use of both WAs and WSMDs.
